# Bilirubin Restrains the Anticancer Effect of Vemurafenib on BRAF-Mutant Melanoma Cells Through ERK-MNK1 Signaling

**DOI:** 10.3389/fonc.2021.698888

**Published:** 2021-06-18

**Authors:** Yufan Tan, Xiaoyu Zhong, Xizhi Wen, Leyi Yao, Zhenlong Shao, Wenshuang Sun, Jiawen Wu, Guanmei Wen, Daolin Tang, Xiaoshi Zhang, Yuning Liao, Jinbao Liu

**Affiliations:** ^1^ Affiliated Cancer Hospital & Institute of Guangzhou Medical University, Guangzhou Municipal and Guangdong Provincial Key Laboratory of Protein Modification and Degradation, School of Basic Medical Sciences, Guangzhou Medical University, Guangzhou, China; ^2^ Institute of Digestive Disease of Guangzhou Medical University, The Sixth Affiliated Hospital of Guangzhou Medical University, Qingyuan People’s Hospital, Qingyuan, China; ^3^ Biotherapy Center, State Key Laboratory of Oncology in South China, Collaborative Innovation Center for Cancer Medicine, Sun Yat-sen University Cancer Center, Guangzhou, China; ^4^ Department of Surgery, The University of Texas Southwestern Medical Center, Dallas, TX, United States

**Keywords:** bilirubin, vemurafenib, ERK, MNK1, BRAF-mutant melanoma

## Abstract

Melanoma, the most threatening cancer in the skin, has been considered to be driven by the carcinogenic RAF-MEK1/2-ERK1/2 signaling pathway. This signaling pathway is usually mainly dysregulated by mutations in BRAF or RAS in skin melanomas. Although inhibitors targeting mutant BRAF, such as vemurafenib, have improved the clinical outcome of melanoma patients with BRAF mutations, the efficiency of vemurafenib is limited in many patients. Here, we show that blood bilirubin in patients with BRAF-mutant melanoma treated with vemurafenib is negatively correlated with clinical outcomes. *In vitro* and animal experiments show that bilirubin can abrogate vemurafenib-induced growth suppression of BRAF-mutant melanoma cells. Moreover, bilirubin can remarkably rescue vemurafenib-induced apoptosis. Mechanically, the activation of ERK-MNK1 axis is required for bilirubin-induced reversal effects post vemurafenib treatment. Our findings not only demonstrate that bilirubin is an unfavorable for patients with BRAF-mutant melanoma who received vemurafenib treatment, but also uncover the underlying mechanism by which bilirubin restrains the anticancer effect of vemurafenib on BRAF-mutant melanoma cells.

## Introduction

Malignant melanoma is one of the most aggressive cancer that initially develops from melanocytes (1), accounting for approximately 90,000 newly diagnosed cases and 10,000 deaths each year (2). Compared with many other cancers, cutaneous melanoma has a particularly poor prognosis and extremely low survival rate due to its high potential of metastasis and a shortage of effectively targeted therapies, leading to a highly undesirable socioeconomic impact for past decades (3, 4).

The highly activated MAPK signaling pathway results from alterations of the BRAF and NRAS genes is the key driver for the development and progression of the majority of cutaneous melanoma (5, 6). Aberrant activation of ERK1/2 results from alterations at multiple levels of the MAPK signaling is also observed in cutaneous melanoma (7). Therefore, MAPK signaling-targeted therapy is gradually being proposed as an important strategy for the treatment of melanoma. The application of vemurafenib, a potent BRAF V600E inhibitor, has provided unprecedentedly therapeutic benefits for patients with cutaneous melanoma over recent years. Nevertheless, acquired resistance to vemurafenib that causes patients to relapse has become a major challenge that needs to be solved urgently (8–10). There are several identified molecular mechanisms underlying the acquired resistance to vemurafenib, including but not limited to NRAS mutation, emergence of BRAF splicing, and BRAF amplification (11–13). However, these mechanisms cannot explain resistance to BRAF inhibitors in approximately 40% of cases (14, 15). Therefore, it is necessary to further eliminate the vemurafenib resistance beyond the genetic modification to finally fight cutaneous melanoma.

Bilirubin is well characterized as a catabolic product of hemoglobin in liver cells. Bilirubin can be conjugated with glucuronic acid in healthy hepatic cells and excreted into the intestine. Under the condition of injury of hepatic cells, bilirubin can be released into the blood and tightly bound to the serum albumin. Therefore, blood bilirubin level is also clinically utilized as a critical predictor for liver function (16). Once bilirubin permeates through the blood brain barrier, it may lead to irreversibly severe encephalic neurotoxicity (17–19). Our recent study shows that interfering the serum bilirubin/albumin concentrations in dementia patients with Aβ deposition through intravenous albumin infusion can improve their clinical outcomes (20). Bilirubin has also been shown to be associated with other diseases, such as cardiovascular protection (21) and obesity reduction (22). Mechanically, bilirubin is recognized as an endogenous antioxidant (23–25) and a potent inhibitor of the proteasomal USP14 and UCHL5 (26).

However, the pathophysiology role and the underlying regulatory mechanism of bilirubin in cancer remain to be elucidated. It is worth to explore the biomarkers and intrinsic factors that influence the efficiency of vemurafenib to improve clinically individual therapy. In this study, our retrospective analysis showed that bilirubin is a critical intrinsic factor that negatively correlates with the efficiency of vemurafenib in patients with BRAF mutant melanoma. Further investigations in cell lines and mouse models demonstrated that bilirubin can potentially reverse vemurafenib-induced proliferation inhibition and apoptosis. Mechanically, we unraveled that the ERK-MNK1 axis is required for bilirubin-induced reversal effects of vemurafenib treatment. These findings not only provide novel insights into the mechanisms underlying vemurafenib resistance, but also may guide the clinical application of vemurafenib for individual anticancer strategies.

## Materials and Methods

### Materials

Bilirubin (B4126-5G) was purchased from Sigma-Aldrich (USA). Vemurafenib (S1267), KO-947 (S8569), and LY3214996 (S8534) were obtained from Selleckchem (Houston, TX). Antibodies, including anti-PARP (#9532), Caspase 3 (#9662), anti-cleaved Caspase-3 (Asp175) (#9661), anti-Caspase 9 (#9502), anti-cleaved Caspase-9 (Asp315) (#9505), anti-phospho-B-Raf (Ser445) (#2696), anti-B-Raf (#14814), anti-phospho-MEK1/2 (Ser217/221) (#3958), anti-MEK1/2 (#4694), anti-phospho-ERK1/2 (Thr202/Tyr204) (#4370), anti-ERK1/2 (#4695), anti-phospho-MNK1 (Thr197/202) (#2111), anti-MNK1 (#2195), anti-phospho-eIF4E (Ser209) (#9741), anti-eIF4E (#2067), anti-phospho-p70S6K (Thr421/Ser424) (#9204), anti-p70S6K (#2708), anti-phospho-4eBP1(Thr37/46) (#2855), anti-4eBP1 (#9644), and anti-GAPDH (#5174) were purchased from Cell Signaling Technology (Beverly, MA).

### Patients

The retrospective study included 28 patients with advanced malignant melanoma who were clinically diagnosed with BRAF V600 mutation in the Affiliated Tumor Hospital of Sun Yat-sen University, and the patients’ informed consent was obtained. These patients were all treated with vemurafenib alone. Among them, 14 were male patients (50%) and 14 were female patients (50%); the median age of the patients was 48.5 years. The blood biochemical data was monitored before and during the treatment, and the medical reference value of each observation index was used as the standard. The average of the upper and lower bounds was taken as the threshold, whereas the clinical outcome was evaluated according to the mRECIST standard and was divided into: complete remission (CR), partial remission (PR), stable disease (SD), and disease progression (PD). Count the number of times each observation index was higher/lower relative to the threshold during the treatment period. The relationship between the level of this indicator and the clinical outcome (CR, PR, SD, and PD) (Spearman rank correlation) was assayed. The outcome variable was defined as ordered level variables assigned the values of 1, 2, 3, and 4, respectively.

### Cell Culture

The human BRAF V600 mutant (A375 and SKMEL28) and wild-type (WM35) melanoma cells were all purchased from ATCC. These cell lines were validated by short tandem repeat profiling, and routine mycoplasma examinations were negative for contamination. These cells were grown in DMEM (Gibco, Invitrogen, Paisley, UK) with 10% FBS in an incubator under the condition of 37°C with 5% CO_2_. The bilirubin treatment was performed at a concentration of 1% FBS.

### RNA Interfering

RNA interfering assay using siRNA transfection was performed as we previously described (27). Briefly, siRNAs targeting MNK1 or control siRNAs were dissolved in RNA free water and stocked at -80°C at a concentration of 10 μM, respectively. A375 and SKMEL28 cells were seeded in 96-well plates or 60 mm dishes. Subsequently, the transfection system was prepared with RPMI opti-MEM (Gibco), lipofectamine™ RNAiMAX (Invitrogen), and siRNAs at a ratio of 500 μl: 10 μl: 10-15 μl. After 10 minutes of incubation, the transfection mixtures were added in the cultured cells at a final concentration of 30-50 nM siRNAs. Further analyses were performed after siRNAs transfection for 48 h.

### Cell Proliferation Assay

Cell proliferation assays, including cell viability and clonogenic assay, were performed according to previous reports (28, 29). Cell viability was determined by the MTS assay (CellTiter 96 Aqueous One Solution reagent). The absorbance of optical density was measured with a microplate reader (Sunrise, Tecan) at wavelength 490 nm. For the clonogenic assay, A375 and SKMEL28 cells were treated with vemurafenib and bilirubin alone or combination, and then suspended in 30% agarose supplemented with 20% FCS and 50% DMEM medium. The resuspended cells were then cultured in 60 mm dishes for 10 days, and then stained with 0.3% crystal violet solution. The colonies > 60 μm were counted under a light microscope. All experiments were done in triplicate.

### Apoptosis Assay

The cell death assay was performed as we previously reported (30). Briefly, A375 and SKMEL28 cells were treated with vemurafenib and bilirubin alone or combination. Cells were digested, harvested, and washed 3 times with cold PBS, followed by resuspending in binding buffer. Then, the Annexin V-FITC and PI solution were added into the cells for 30 min in the dark. Finally, the cells were analyzed by flow cytometry or kinetically imaged with an inverted fluorescence microscope equipped with a digital camera (AxioObsever Z1, Zeiss). All experiments were done in triplicate.

### Western Blotting

This assay was performed after the protein preparation and protein determination at 4°C as we previously reported (31, 32). Blue SDS loading buffer was added into the protein solution and subjected to a boiling water bath for 5 minutes. The protein was concentrated by 5% SDS-PAGE for 30 min, separated by 12% SDS-PAGE for 1 h, and then transferred to a PVDF membrane. 5% non-fat milk was utilized to block the membrane for 1 h at room temperature. The membrane was incubated with indicated primary antibodies at 4°C overnight, and then subjected to secondary antibodies at room temperature for 1 h. Finally, the ECL detection reagents (Thermo Scientific) were used to link to the secondary antibodies and react to X-ray films.

### Animal Study

SPF grade BALB/c male nude mice weighting about 20-22g (Guangdong Provincial Animal Experiment Center) were bred in the Animal Center of Guangzhou Medical University. This study was approved by Institutional Animal Care and Use Committee. After 3 days, 1×10^6^ A375 cells were subcutaneously inoculated into the right axilla of BALB/c nude mice to establish the xenograft model as we previously reported (33). Then the mice were randomly divided into 4 groups when the tumor volume reached 80-100 mm^3^. Bilirubin (20 mg/kg/d) was administered by intraperitoneal injection, whereas vemurafenib was administered by oral gavage (75 mg/kg/d). The body weight and tumor size of nude mice were measured every other day. After administration for 12 days, the nude mice were sacrificed by cervical dislocation post CO_2_ inhalation.

### Statistical Analysis

Data were analyzed using SPSS 16.0 and GraphPad Prism 7 software and presented as mean and standard deviation (SD). To determine statistical probabilities, an unpaired Student’s t-test or one-way ANOVA was conducted where appropriate. The rank correlation was subjected to Spearman rank analysis. *P <*0.05 was considered statistically significant.

## Results

### Clinical Relationship Between Blood Bilirubin-Related Markers and Outcome in BRAF Mutant Melanoma

**Figure 1:**
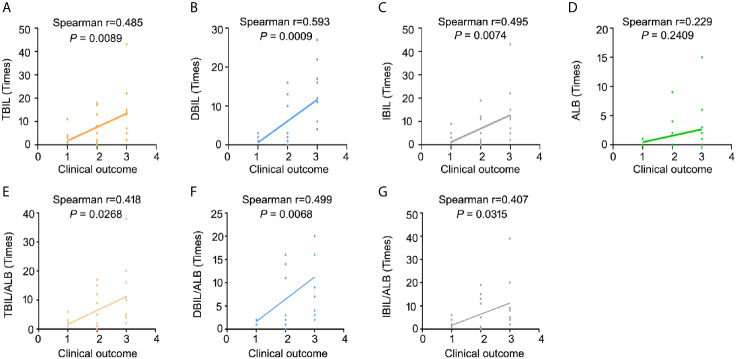
Relationship between blood bilirubin and clinical outcome in patients with BRAF mutant melanoma. Statistical analysis of blood biochemical data during hospitalization of 28 patients with BRAF V600 mutant malignant melanoma who received vemurafenib alone. **(A)** Spearman correlation analysis between the time of TBIL below the threshold and the clinical outcome. **(B)** Spearman correlation analysis between the time of DBIL below the threshold and the clinical outcome. **(C)** Spearman correlation analysis between the time of IBIL below the threshold and the clinical outcome. **(D)** Spearman correlation analysis between the time of ALB higher than the threshold and the clinical outcome. **(E–G)** Spearman correlation analysis between the time of TBIL/ALB below the threshold and the clinical outcome, DBIL/ALB below the threshold and the clinical outcome, and IBIL/ALB below the threshold and the clinical outcome.

Despite the significance of bilirubin has been gradually recognized in several diseases, the role of bilirubin in the treatment of vemurafenib in BRAF mutant melanoma is still unclear. To investigate the relationship between blood bilirubin-related markers and clinical outcome of patients with BRAF mutant melanoma, we first conducted a clinically retrospective analysis of 28 patients with advanced malignant melanoma who were diagnosed with BRAF V600 mutation and were treated with vemurafenib alone in the Affiliated Tumor Hospital of Sun Yat-sen University. There was no correlation between age/gender and clinical outcome of patients with BRAF V600 mutation **(** **Figure S1** **)**. The results showed that the time of total bilirubin (TBIL), direct bilirubin (DBIL), or indirect bilirubin (IBIL) below the threshold level was positively correlated with the clinical outcome of these patients **(** **Figures 1A–C** **)**. In contrast, the time of albumin (ALB) higher than the threshold level was not significantly related to the clinical outcome of patients **(** **Figure 1D** **)**. Additionally, the time of TBIL/ALB, DBIL/ALB, or IBIL/ALB below the threshold level was also positively correlated with the clinical outcome of patients **(** **Figures 1E–G** **)**. These findings demonstrate that blood bilirubin not only predicts the poor outcome for melanoma patients, but also may act as a negative factor in the treatment of BRAF mutant melanoma with vemurafenib.

### Bilirubin Abrogates Vemurafenib-Induced Growth Inhibition of Melanoma Cells *In Vitro* and *In Vivo*

**Figure 2:**
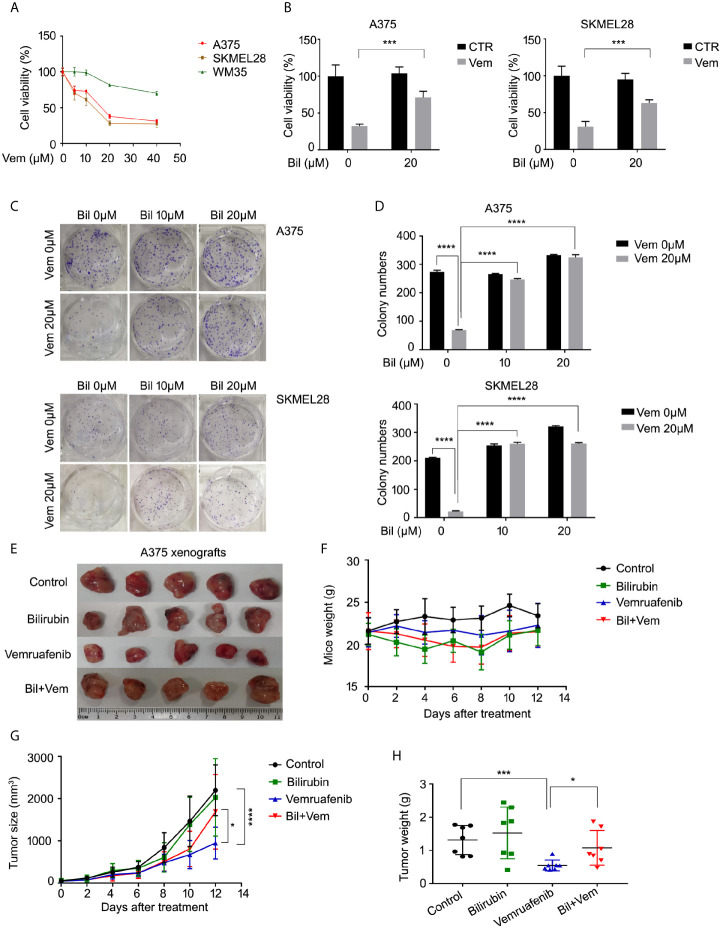
Bilirubin abrogates vemurafenib-induced proliferation suppression in BRAF mutant melanoma cell lines and xenografts. **(A)** A375, SKMEL28, and WM35 cells were treated with vemurafenib (Vem) for 24 h. Cell viability was determined using MTS assay for triplicate. Mean±SD (n = 3). **(B)** Cell viability of A375 and SKMEL28 cells following treatment with Vem with or without bilirubin (Bil) for 24 h for triplicate. Mean± SD (n = 3). **(C)** Colony formation assay of A375 and SKMEL28 cells following treatment with Vem with or without Bil for 24 h. **(D)** Quantification of colony formation **(C)** was shown. Mean±SD (n = 3). **(E)** Images of A375 xenografts from nude mice treated with Vem (75 mg/kg/d), Bil (20 mg/kg/d), or Bil+Vem for 12 days. **(F)** Body weight of mice, **(G)** tumor size, and **(H)** tumor weight was shown. *P < 0.05, ***P < 0.001, ****P < 0.0001.

Since the level of bilirubin was negatively correlated with the clinical outcome of patients who were treated with vemurafenib, we wondered whether bilirubin could abrogate the anti-cancer effects of vemurafenib in BRAF mutant melanoma models. First, we validated the cell proliferative inhibition of vemurafenib in two BRAF V600 mutant cell lines (A375 and SKMEL28) and the wide-type cell line WM35. The results showed that BRAF V600 mutant cell lines were more sensitive to the treatment of vemurafenib than the wide-type cell line **(** **Figure 2A** **)**. Subsequently, we determined the cell viability of A375 and SKMEL28 cells following vemurafenib treatment in the absence or presence of bilirubin. We found that bilirubin significantly reversed vemurafenib-induced cell viability inhibition in two BRAF V600 mutant cell lines **(** **Figure 2B** **)**. Additionally, the colony formation assay showed that vemurafenib-induced long-term proliferative suppression was rescued by the treatment of bilirubin **(** **Figures 2C, D** **)**. To further address whether bilirubin could abrogate anti-cancer effects of vemurafenib *in vivo*, BALB/c nude mice models were established and treated with vemurafenib and bilirubin alone or in combination for 12 days. The results showed that the tumor size and weight of the vemurafenib treatment group were much lower than those of the control group, and this anti-tumor effect *in vivo* was reversed by bilirubin treatment **(** **Figures 2E–H** **)**. Taken together, these findings demonstrate that bilirubin can effectively block the growth inhibition effects of vemurafenib on BRAF V600 mutant melanoma in cell lines and xenograft models.

### Bilirubin Reverses Vemurafenib-Induced Apoptosis

**Figure 3:**
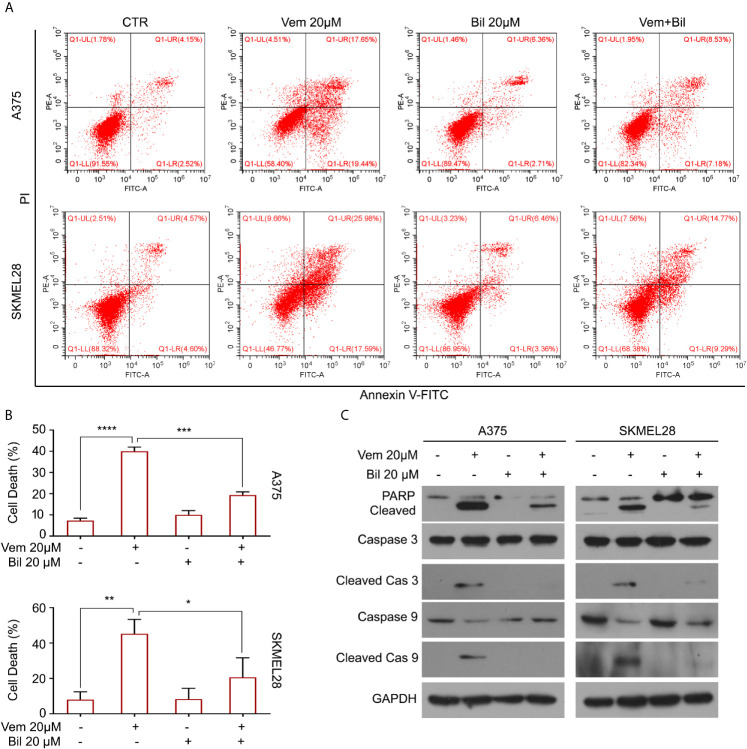
Bilirubin reverses vemurafenib-induced apoptosis in melanoma cells. **(A)** A375 and SKMEL28 cells were treated with Vem, Bil, and V+B for 18 h. Cells were stained with Annexin V-FITC and PI for 30 min and subjected to flow cytometry analysis. Representative images were shown. **(B)** Quantification of cell death in **(A)** from triplicate was shown. Mean±SD (n = 3). *P < 0.05, **P < 0.01, ***P < 0.001, ****P < 0.0001. **(C)** A375 and SKMEL28 cells were treated with Vem, Bil, and V+B for 24 h. Western blot assay was performed using PARP, cleaved caspase 3, caspase 3, cleaved caspase 9, caspase 9, and GAPDH antibodies.

**Figure 4:**
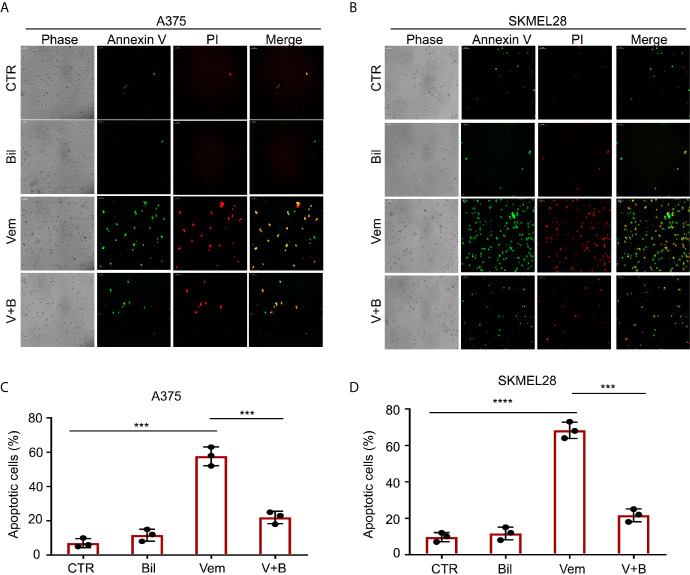
Morphological findings of bilirubin reversal of vemurafenib-induced apoptosis. A375 and SKMEL28 cells were treated with Vem, Bil, and V+B for 24 h. Cells were stained with Annexin V-FITC and PI for 30 min. **(A, B)** Representative images of Annexin V-FITC-positive (green) and PI-positive (red) A375 and SKMEL28 cells were shown. **(C, D)** Quantification of Annexin V-FITC-positive (green) and PI-positive (red) A375 and SKMEL28 cells from triplicate was shown. Mean±SD (n = 3). ***P < 0.001, ****P < 0.0001.

Cell apoptosis is a key biological process in the inhibition of proliferation of BRAF V600 mutant melanoma induced by vemurafenib. Hence, we wondered whether bilirubin may alter the percentage of vemurafenib-induced apoptosis. The Annexin V-FITC and propidium iodide (PI) double staining followed by flow cytometry analysis was performed in A375 and SKMEL28 cells treated with vemurafenib and bilirubin alone or in combination. As expected, bilirubin reversed vemurafenib-induced apoptosis in BRAF V600 mutant melanoma cells **(** **Figures 3A, B** **)**. Next, we examined apoptosis-driven molecular events, such as the expression of activated caspase 9, activated caspase 3, and the cleavage form of PARP, in A375 and SKMEL28 cells following treatment with vemurafenib and bilirubin alone or in combination. The results of western blotting showed that bilirubin markedly reversed vemurafenib-induced the activation of caspase 9, caspase 3, and PARP cleavage **(** **Figure 3C** **)**. To observe the morphological changes, we further performed Annexin V-FITC and PI double staining and subjected to an inverted fluorescence microscope in A375 and SKMEL28 cells. Indeed, additional morphological evidence confirmed that bilirubin is able to block vemurafenib-induced apoptosis **(** **Figures 4A–D** **)**. Taken together, these findings illustrate that bilirubin reversed vemurafenib-induced proliferation suppression mainly by blocking apoptosis in BRAF V600 mutant melanoma cells.

### Bilirubin Reactivates ERK Signaling After Vemurafenib Treatment

**Figure 5:**
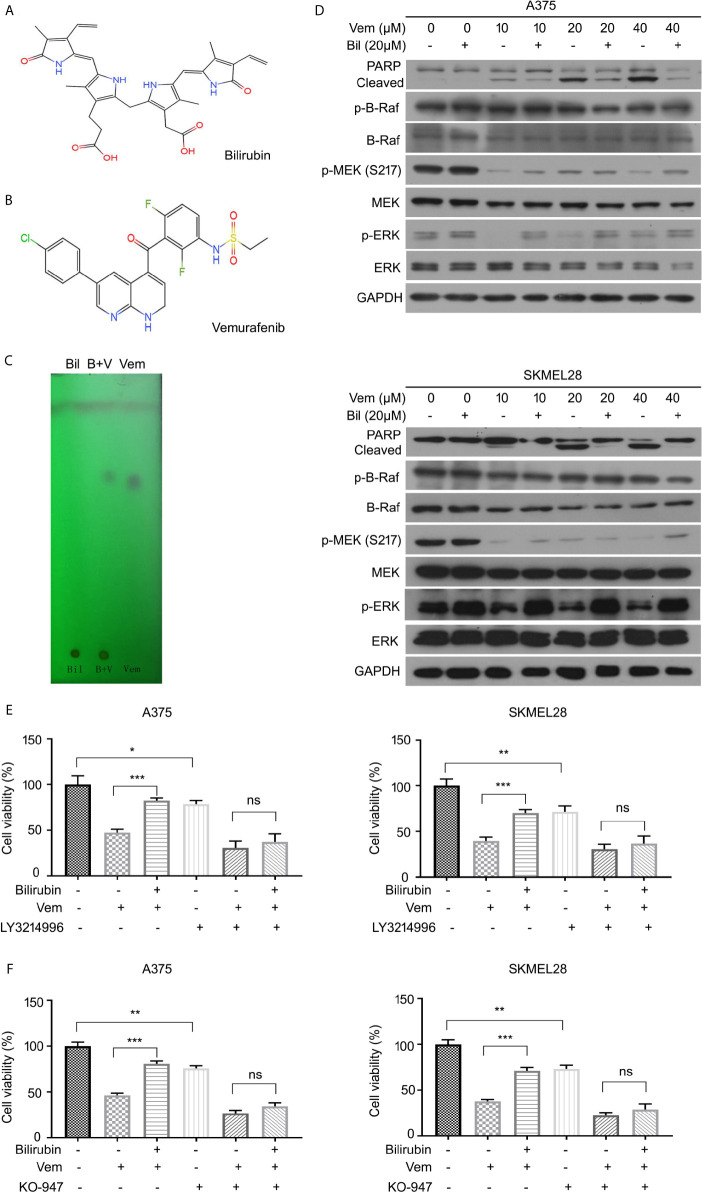
Bilirubin reactivates ERK phosphorylation after vemurafenib treatment in melanoma. **(A, B)** Chemical structural formula of Bil and Vem. **(C)** Determination of the polarities of Vem, Bil, and V+B by adsorption thin layer chromatography. **(D)** A375 and SKMEL28 cells were treated with indicated doses of Vem, Bil, and V+B for 24 h. Western blot was performed using PARP, p-B-Raf, B-Raf, p-MEK, MEK, p-ERK, ERK, and GAPDH antibodies. **(E, F)** A375 and SKMEL28 cells were treated with Vem, Bil, or V+B for 24 h with or without LY3214996 (10 μM) or KO-947 (2 μM). Cell viability was performed for triplicate. Mean±SD (n = 3). *P < 0.05, **P < 0.01, ***P < 0.001, ns, not significant.

To further explore the underlying molecular mechanism by which bilirubin reversed vemurafenib-induced proliferation inhibition, we first determined whether it may involve the potential chemical-chemical interactions. Determination of the polarities of vemurafenib and bilirubin alone or in combination was performed using adsorption thin layer chromatography. This assay showed that vemurafenib does not interact with bilirubin **(** **Figures 5A–C** **)**, indicating that chemical-chemical interaction is not involved in this process. Next, we determined the activation of B-Raf-MEK1/2-ERK1/2 signaling pathway by detecting the expression of phospho-B-Raf, phospho-MEK1/2, and phospho-ERK1/2 in A375 and SKMEL28 cells treated with vemurafenib in the absence or presence of bilirubin. Western blot analysis showed that bilirubin reactivated ERK1/2, but not B-Raf and MEK1/2, in A375 and SKMEL28 cells **(** **Figure 5D** **)**. To determine whether the reactivation of ERK1/2 is required for bilirubin reversed proliferation inhibition induced by vemurafenib, we used two specific inhibitors of ERK1/2, LY3214996 and KO-947. Bilirubin failed to reverse the proliferation inhibition induced by vemurafenib after the pharmacological inhibition of ERK1/2 by LY3214996 or KO-947 **(** **Figures 5E, F** **)**. Collectively, reactivation of ERK1/2 signaling is required for bilirubin reversed vemurafenib-induced proliferation inhibition.

### The ERK-MNK1 Axis Plays a Critical Role in Bilirubin-Induced Reversal Effects in Melanoma Cells With BRAF Mutants

**Figure 6:**
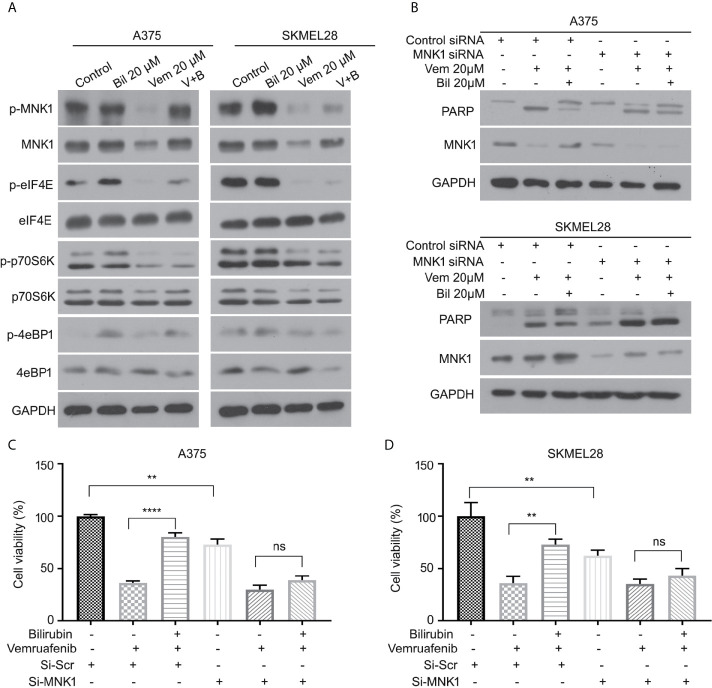
ERK-MNK1 signaling is required for bilirubin-induced reversal effects after vemurafenib treatment. **(A)** A375 and SKMEL28 cells were treated with Vem, Bil, or V+B for 24 h. Western blot was performed using p-MNK1, MNK, p-eIF4E, eIF4E, p-p70S6K, p70S6K, p-4eBP1, 4eBP1, and GAPDH antibodies. **(B)** A375 and SKMEL28 cells were treated with Vem, Bil, or V+B for 24 h with or without the transfection of MNK1 siRNAs for 48 h. Western blot was performed using PARP, MNK1, and GAPDH antibodies. **(C, D)** A375 and SKMEL28 cells were treated with Vem, Bil, or V+B for 24 h with or without the transfection of MNK1 siRNAs for 48 h. Cell viability was performed for triplicate. Mean±SD (n = 3). **P < 0.01, ****P < 0.0001, ns, not significant.

We further determined the expression of several key downstream regulators of ERK1/2 (e.g., MNK1, eIF4E, p70S6K, and 4eBP1) in A375 and SKMEL28 cells in response to vemurafenib with or without bilirubin. Bilirubin only reversed the expression levels of phospho-MNK1 and pan-MNK1, but not other downstream regulators **(** **Figure 6A** **)**. To investigate the role MNK1 in regulating bilirubin activity, we determined the expression of PARP cleavage in wild type and MNK1-knockdown A375 and SKMEL28 cells following treatment with vemurafenib and bilirubin. Of note, bilirubin failed to reverse the expression of PARP cleavage triggered by vemurafenib after the depletion of MNK1 by siRNAs in BRAF mutant melanoma cells **(** **Figure 6B** **)**. Our cell viability assay also showed that bilirubin failed to rescue vemurafenib-triggered proliferation suppression **(** **Figures 6C, D** **)**. Altogether, these findings demonstrate that the ERK1/2-MNK1 axis is critical to bilirubin-induced blockage of vemurafenib in BRAF V600 mutant melanoma cells.

## Discussion

Acquired or adaptive resistance to vemurafenib is a challenge in melanoma therapy. Although several molecular mechanisms underlying the resistance to vemurafenib have been identified, they hardly cover all cases of patients with vemurafenib resistance. In this study, our retrospective analysis found that the time of TBIL, DBIL, or IBIL below the threshold levels was positively correlated with the clinical outcome of patients with BRAF mutant melanoma who received only vemurafenib therapy. Further experiments in cell lines and nude mice bearing xenografts of BRAF mutant melanoma confirmed that bilirubin is able to strongly restrain the anticancer effect of vemurafenib. More importantly, we unraveled that the ERK1/2-MNK1 axis is required for bilirubin-induced reversal of apoptosis and growth suppression triggered by vemurafenib. These findings may enrich our understanding of the microenvironmental factors underlying vemurafenib resistance and provide experimental evidence for clinical guidelines.

Bilirubin has been considered as a catabolic waste of hemoglobin for a long time. Despite the increase in biological activities (such as antioxidation and proteasome inhibition) in certain models has been established (17, 26), the roles of bilirubin in various diseases are still mysterious. Our previous study has been demonstrated that bilirubin-induced encephalopathy in newborn rats leads to protein hyperphosphorylation of Tau and Aβ accumulation, thereby inducing AD-like pathological alterations in their later life (19). Subsequent study has been shown that interfering with the ratio of serum bilirubin/albumin by albumin infusion improves the outcome of dementia patients with the deposition of Aβ (20). For the treatment of BRAF-mutant melanoma, the drug instructions have clearly pointed out that vemurafenib can cause abnormal liver function. Therefore, liver enzymes and bilirubin in patients must be monitored monthly during the treatment with vemurafenib. In this study, we wondered whether bilirubin may influence the clinical outcome of patients with BRAF mutant melanoma. Our current retrospective study showed that not only the time of TBIL, DBIL, and IBIL, but also TBIL/ALB, DBIL/ALB and IBIL/ALB below the threshold levels, were positively correlated with their clinical outcome after vemurafenib treatment. These findings not only indicate that bilirubin is a critical microenvironmental factor that negatively affects the efficacy of vemurafenib in patients with BRAF mutant, but also suggest that the melanoma patients with jaundice may reduce the efficacy of vemurafenib and predict poor prognosis. Thus, our study may provide a basis for guiding the clinical application of vemurafenib in patients with melanoma.

Our clinical observations on the relationship between blood bilirubin and clinical outcome of patients treated with vemurafenib further prompted us to explore the effect of bilirubin on vemurafenib activity *in vitro* and *in vivo*. Consistently with the retrospective findings, we found that bilirubin significantly inhibited the antitumor effect of vemurafenib in BRAF mutant melanoma cell lines and xenografts. Since vemurafenib can inhibit the growth of BRAF mutant melanoma cells *via* apoptosis induction (34, 35), we explored whether apoptosis is involved in bilirubin-induced reversal effect of proliferation suppression triggered by vemurafenib. Our flow cytometry and immunoblot assays collectively confirmed that bilirubin effectively rescues caspase-mediated apoptosis induced by vemurafenib in both A375 and SKMEL28 cells. Thus, bilirubin restrains the antitumor effects of vemurafenib by inhibiting vemurafenib-induced apoptosis, and further confirmed the rationality and causality of our clinically retrospective observations.

In regard to exploring the molecular mechanisms of bilirubin inhibiting the efficacy of vemurafenib in the treatment of melanoma, this study first excluded the chemical-chemical interaction between bilirubin and vemurafenib through an adsorption thin layer chromatography assay. On the contrary, bilirubin may reverse the phosphorylation level of ERK, but not the phosphorylation of BRAF and MEK. Additionally, the pharmacological inhibition of ERK1/2 with LY3214996 or KO-947 significantly abrogates bilirubin-induced reversal effects of growth suppression triggered by vemurafenib. These results were partly consistent with previous findings that the MEK/ERK axis is involved in vemurafenib resistance in BRAF mutant melanoma (36, 37). MNK1, eIF4E, p70S6K, and 4eBP1 are key downstream effectors of ERK1/2 (38–41). Notably, this study unravels that MNK1 is the key effector responsible for the bilirubin-induced reversal effects due to the following findings: 1) bilirubin can reverse the downregulation of MNK1 and phosphorylated MNK1, but not other effectors triggered by vemurafenib; 2) bilirubin failed to display the reversal effects of growth inhibition and apoptosis induction triggered by vemurafenib after MNK1 depletion. Collectively, we proposed that the ERK1/2-MNK1 axis is required for bilirubin-induced reversal of growth inhibition and apoptosis induction in BRAF mutant melanoma following vemurafenib treatment.

Overall, our current study reveals that bilirubin is a microenvironmental factor that can inhibit the activity of vemurafenib *via* reactivation of the ERK1/2-MNK1 signaling, which may help to develop new anti-tumor strategies in patients with BRAF mutant melanoma.

## Data Availability Statement

The original contributions presented in the study are included in the article/**Supplementary Material**. Further inquiries can be directed to the corresponding authors.

## Ethics Statement

The animal study was reviewed and approved by The ethics committee of Guangzhou Medical University. Written informed consent was obtained from the individual(s) for the publication of any potentially identifiable images or data included in this article.

## Author Contributions 

JL, YT, GW and YL designed experiments. YT, XW and XSZ performed the correlation analysis in retrospective study. YT, XYZ, LY, ZS, JW, and WS performed laboratory experiments. JL, YL and DT wrote and revised the manuscript. All authors contributed to the article and approved the submitted version. YT, XYZ and XW contributed equally to this work.

## Funding

This work was supported by National Natural Science Foundation of China (81773213 and 81972399), Natural Science Foundation of Guangdong Province (2018A0303130024), Natural Science Foundation research team of Guangdong Province (2018B030312001), and the open research funds from the Sixth Affiliated Hospital of Guangzhou Medical University, Qingyuan People’s Hospital (202011-204 and 202011-106).

## Conflict of Interest

The authors declare that the research was conducted in the absence of any commercial or financial relationships that could be construed as a potential conflict of interest.
