# Supplementary material for: Bilirubin Restrains the Anticancer Effect of Vemurafenib on BRAF-Mutant Melanoma Cells Through ERK-MNK1 Signaling
Source: Front Oncol. 2021 Jun 18;11:698888. doi: 10.3389/fonc.2021.698888 (PMC8250144; doi:10.3389/fonc.2021.698888)
Supplement: Supplementary file 1 [file DataSheet_1.pdf]

1.Western blots

Fig.3C

A375 cells

PARP

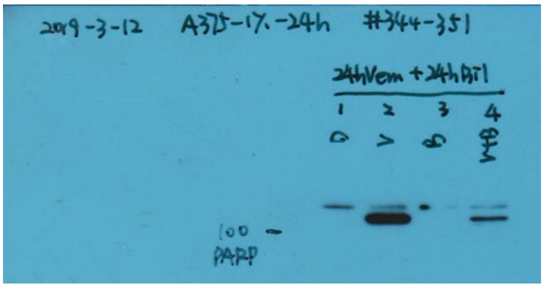

Caspase 3

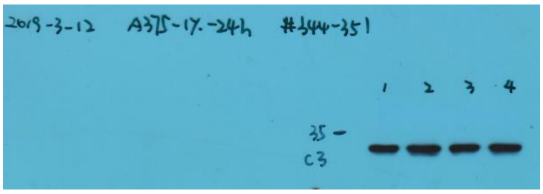

Cleaved Cas 3

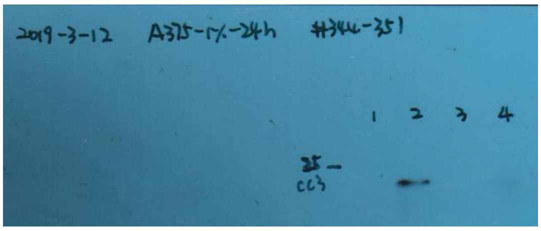

Caspase 9

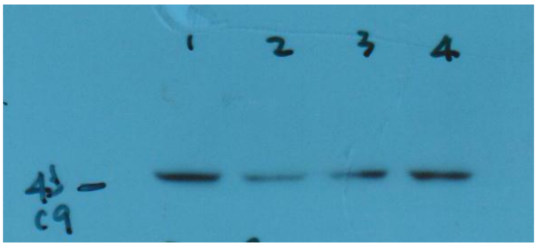

Cleaved Cas 9

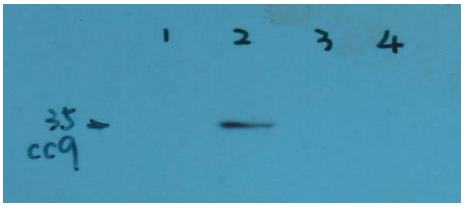

GAPDH

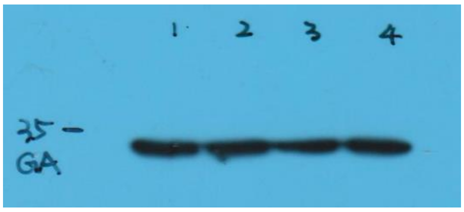

SKMEL28 cells

PARP

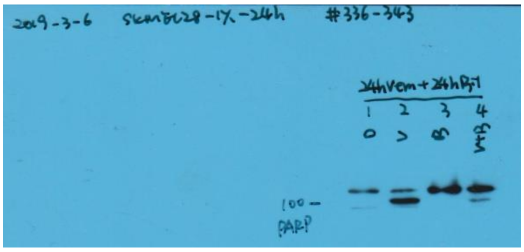

Caspase 3

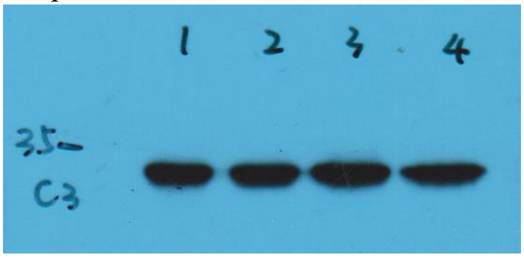

Cleaved Cas 3

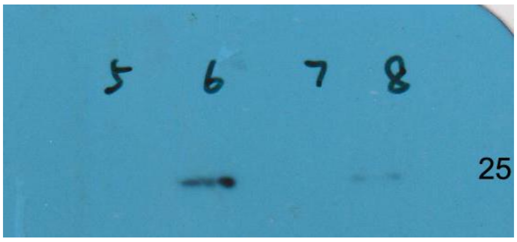

Caspase 9

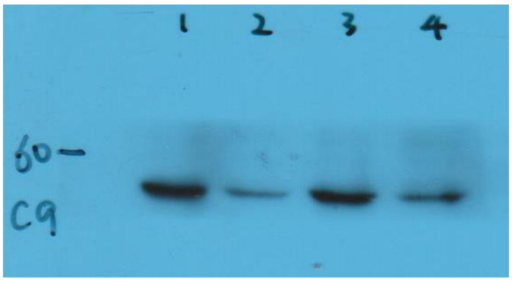

Cleaved Cas 9

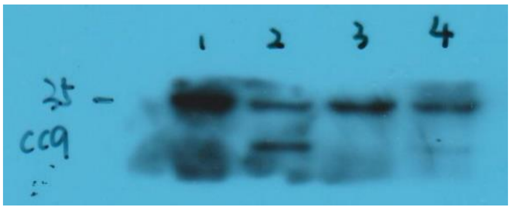

GAPDH

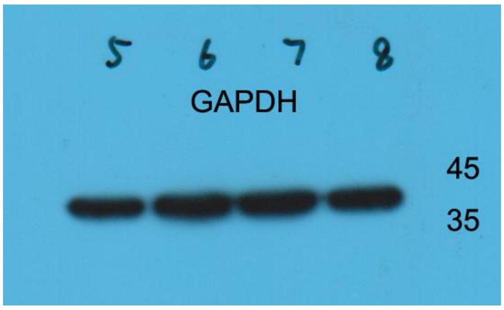

Fig.5D

A375 cells

PARP

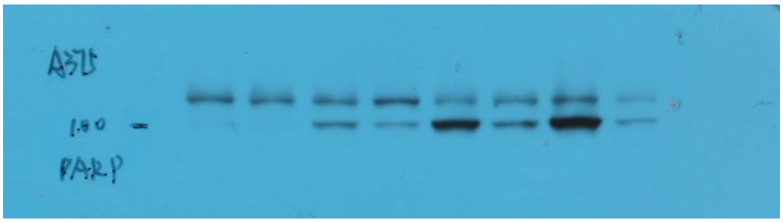

p-B-Raf

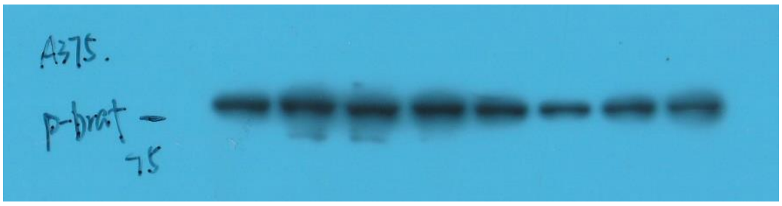

B-Raf

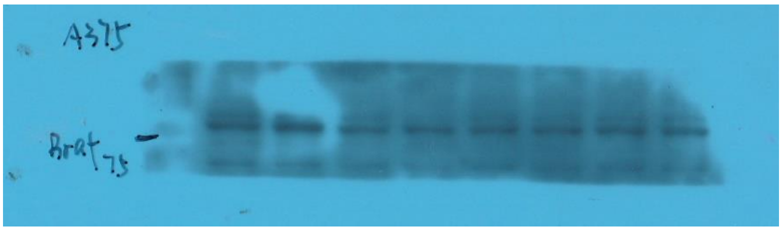

p-MEK

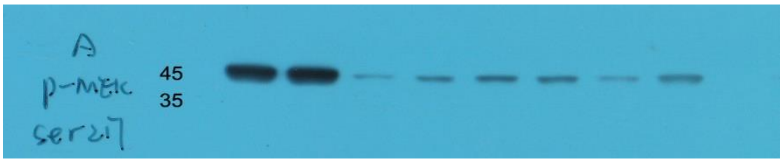

p-ERK

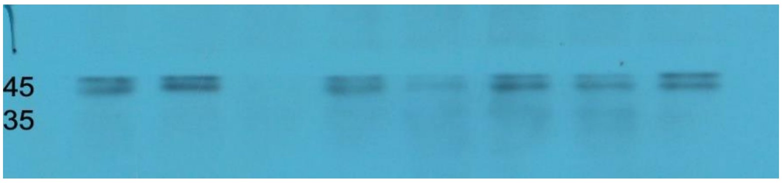

ERK

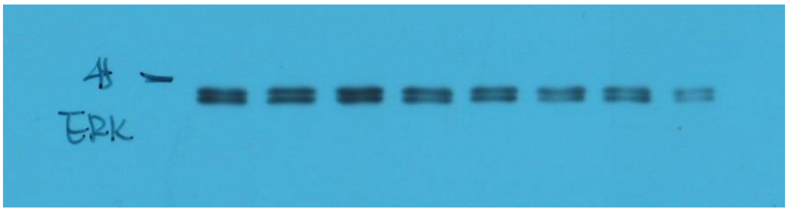

GAPDH

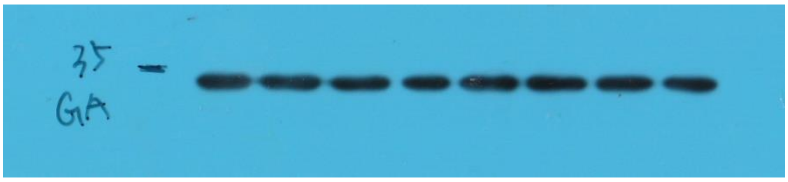

Fig.5D

SKMEL28 cells

PARP

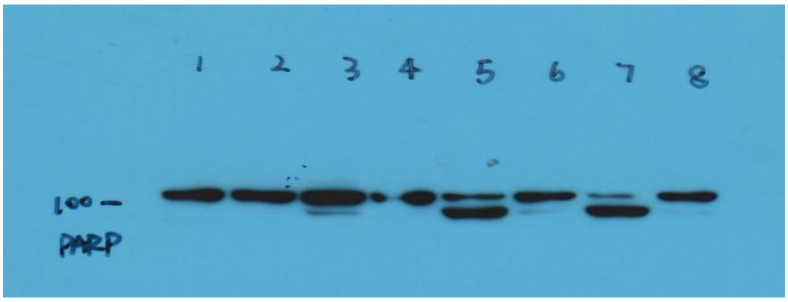

p-B-Raf

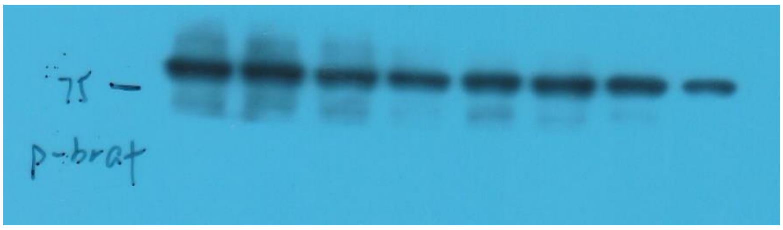

B-Raf

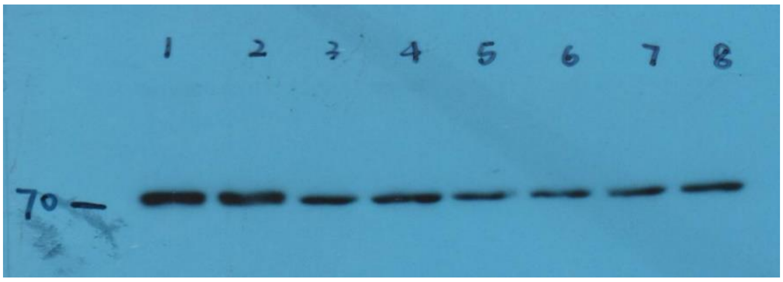

p-MEK

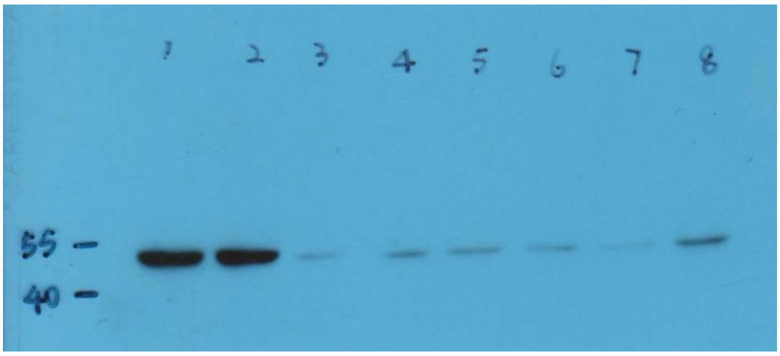

MEK

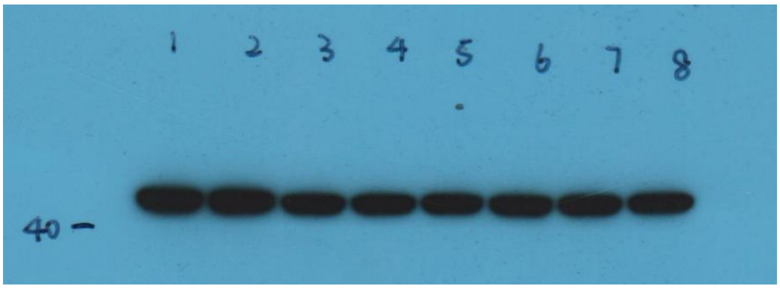

p-ERK

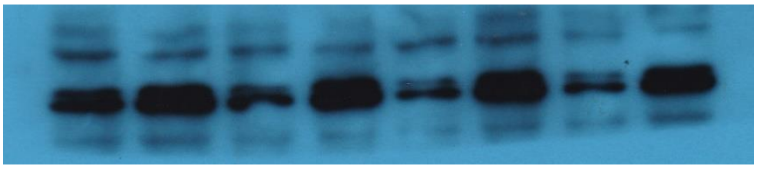

ERK

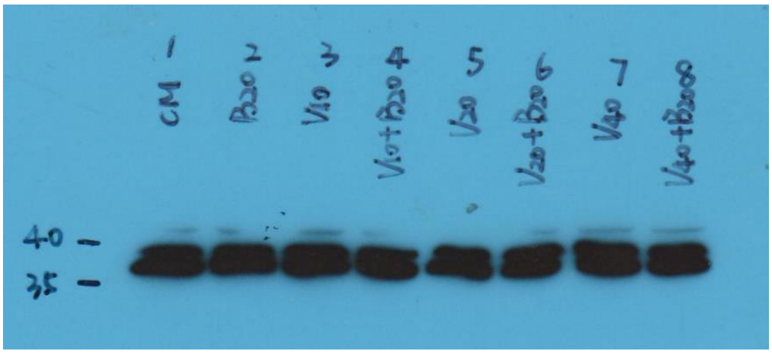

GAPDH

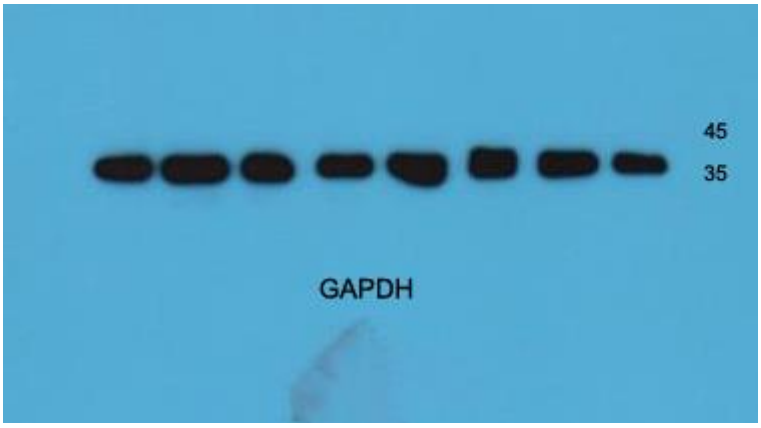

Fig.6A

A375 cells

p-MNK1

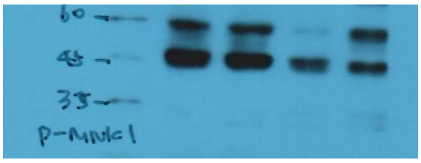

MNK1

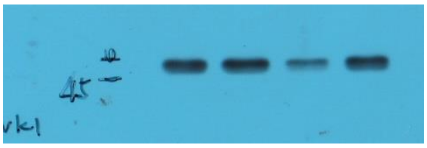

p-eIF4E

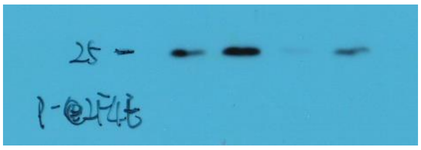

e-IF4E

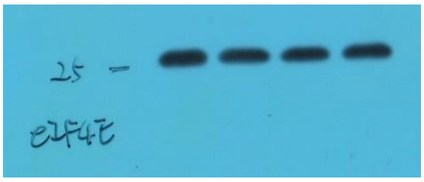

p-p70S6K

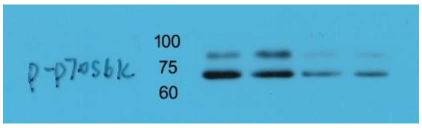

p70S6K

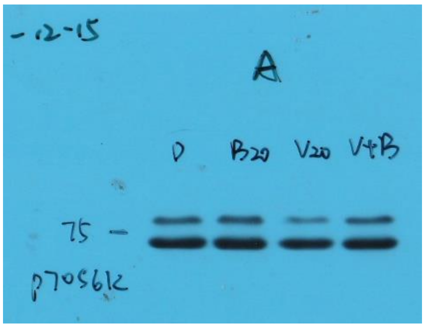

p-4eBP1

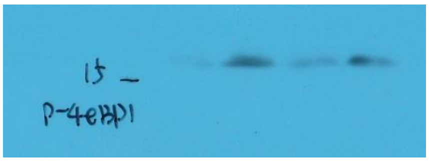

4eBP1

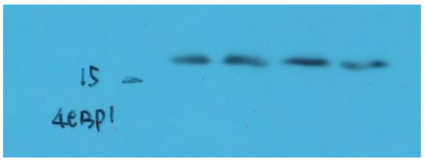

GAPDH

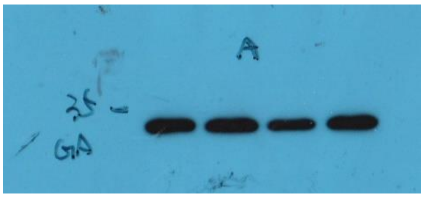

Fig.6A
SKMEL28 cells
p-MNK1

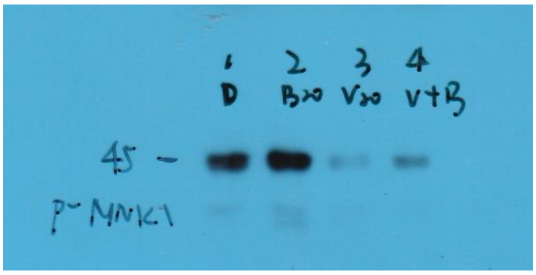

MNK1

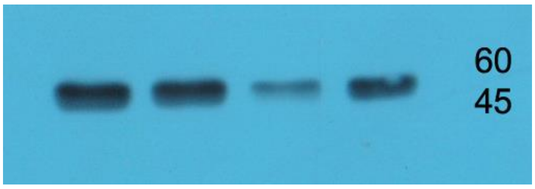

p-eIF4E

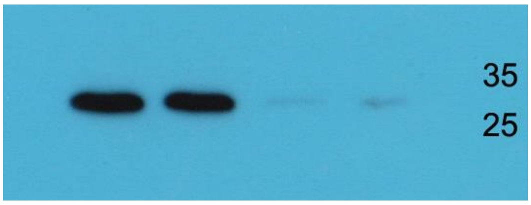

eIF4E

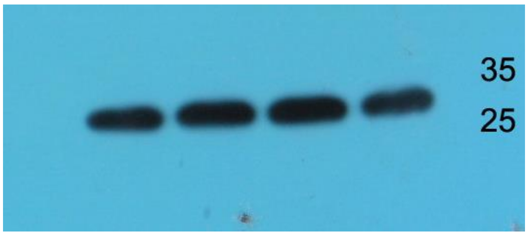

p-p70S6K

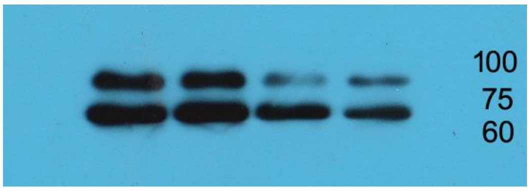

p70S6K

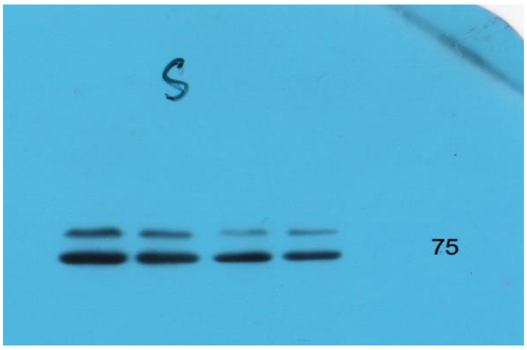

p-4eBP1

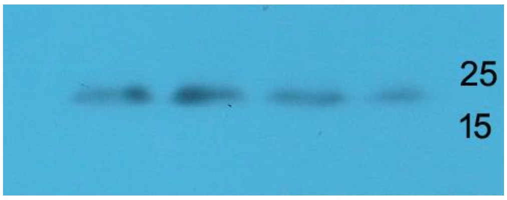

4eBP1

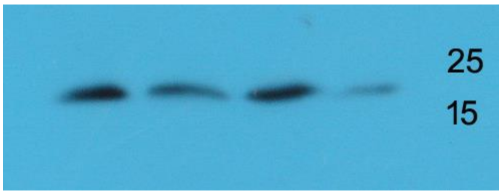

GAPDH

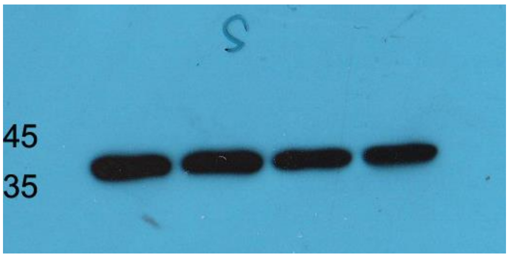

Fig.6B

A375 cells

PARP

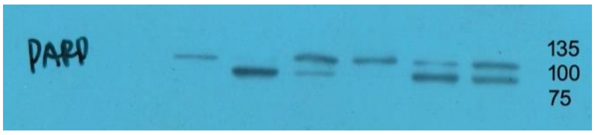

MNK1

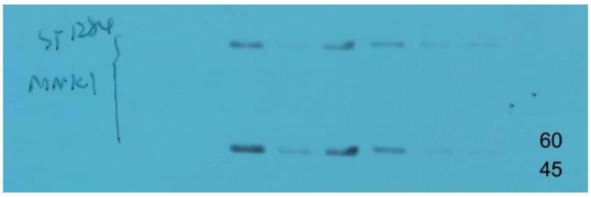

GAPDH

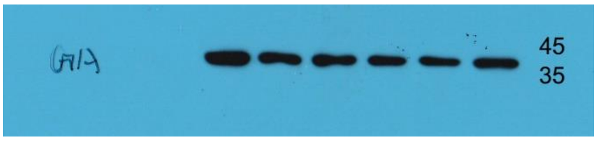

SKMEL28 cells

PARP

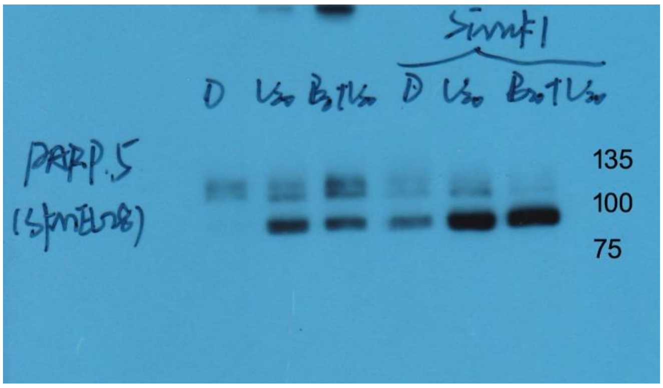

MNK1

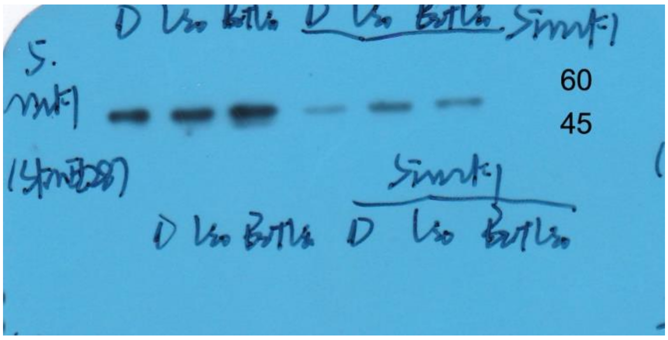

GAPDH

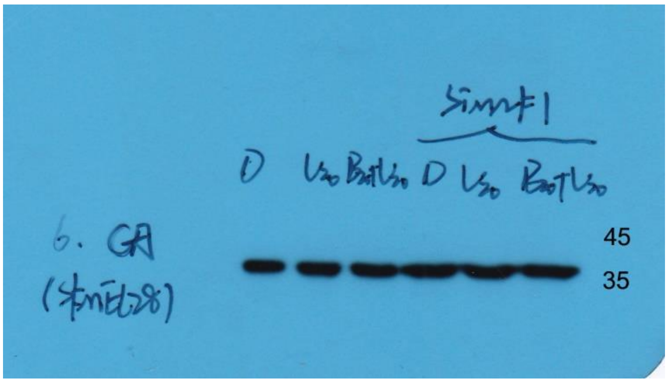

2.Flowcytometry images

Fig.3A

A375 cells

CTR

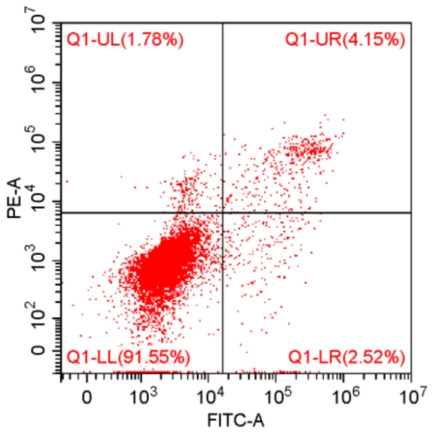

Vem 20μM

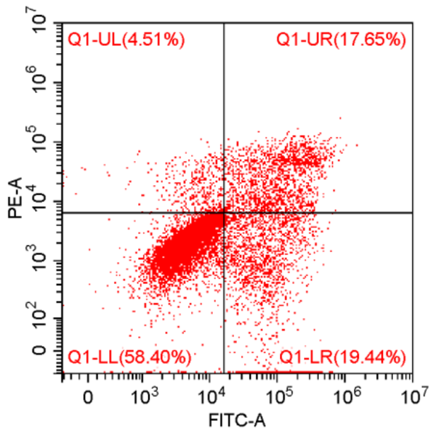

Bil 20μM

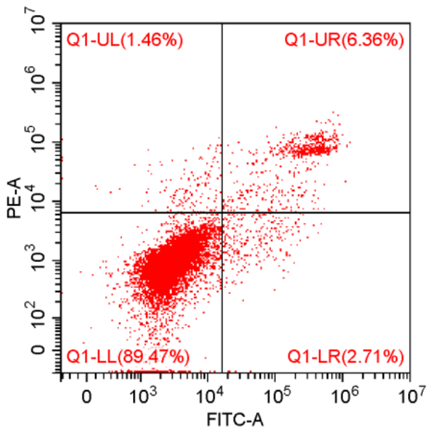

V+B

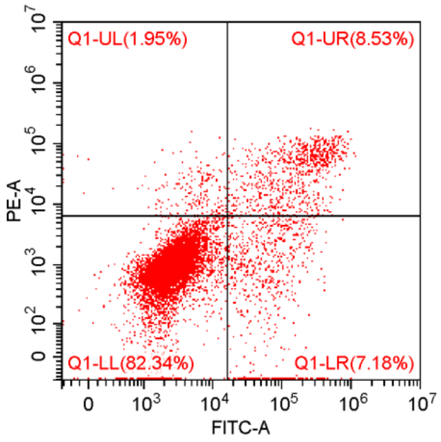

Fig.3A

SKMEL28 cells

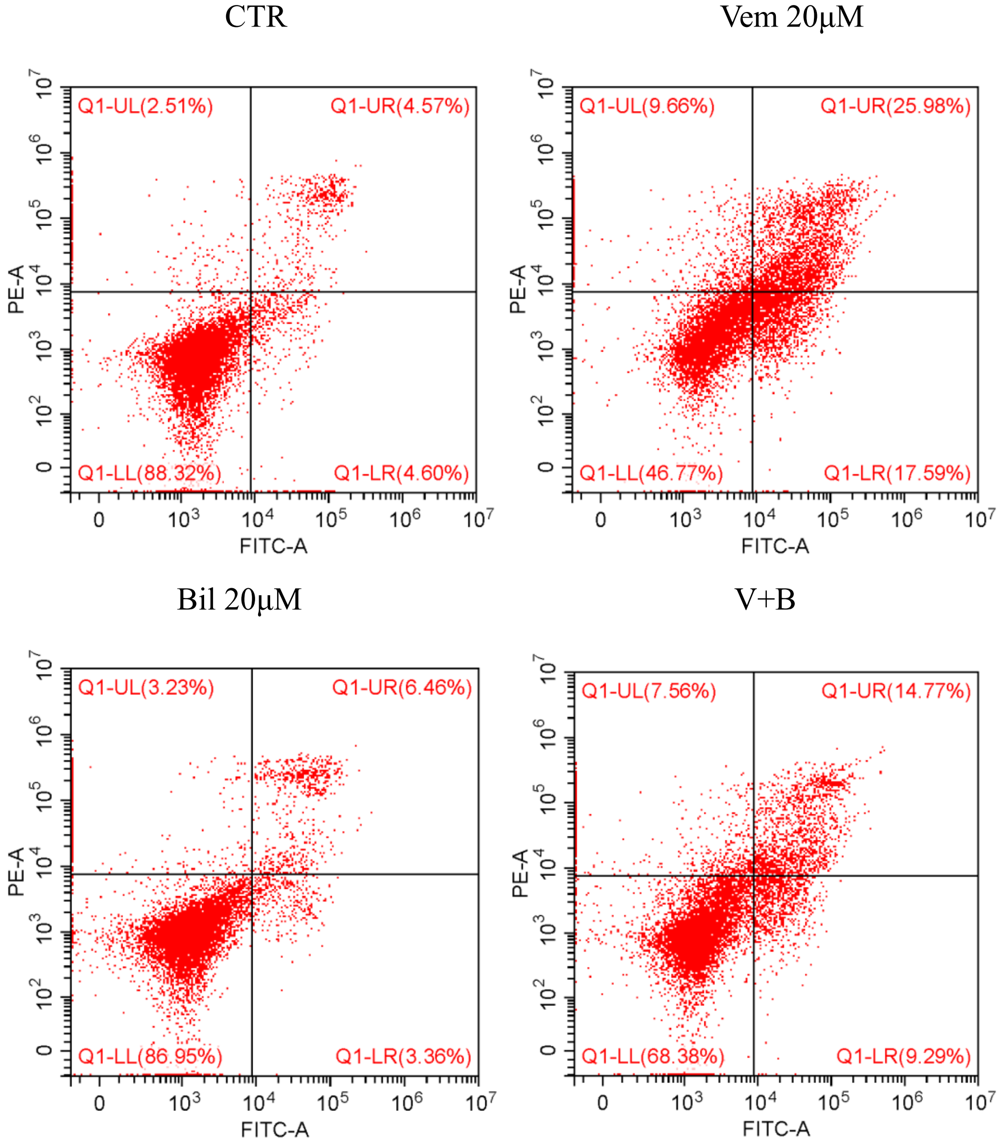

3.Microscopy images

Fig.4A-A375 cells

CTR-PH

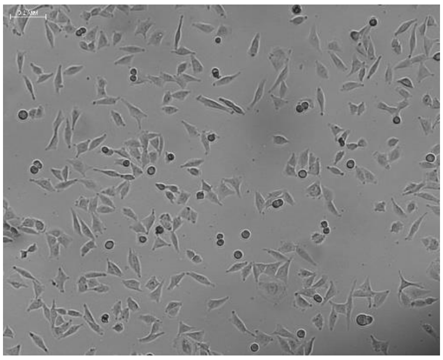

CTR-Annexin V-FITC

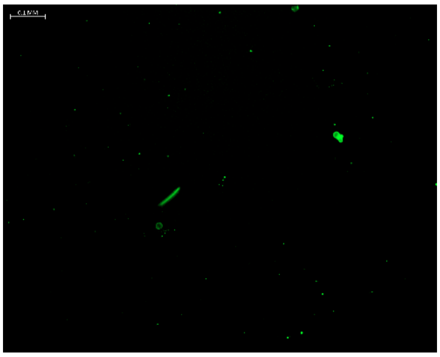

CTR-PI

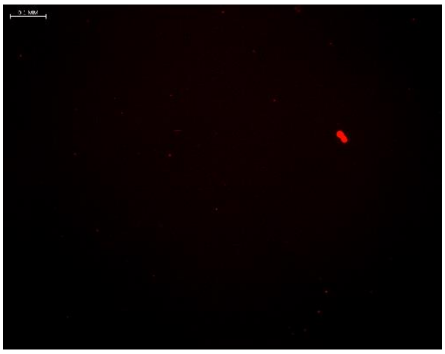

CTR-Merge

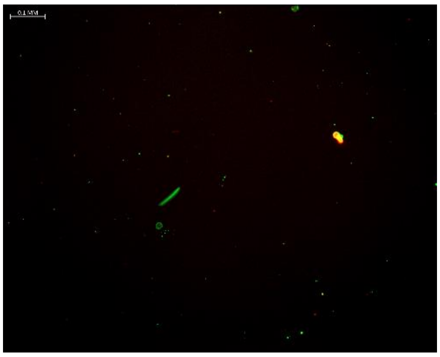

Bil-PH

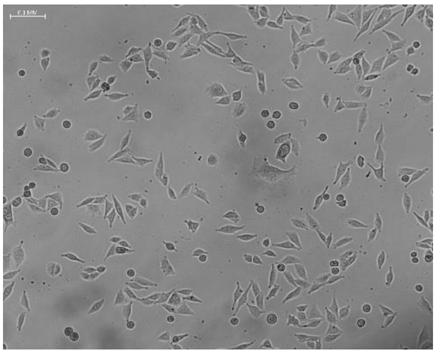

Bil-Annexin V-FITC

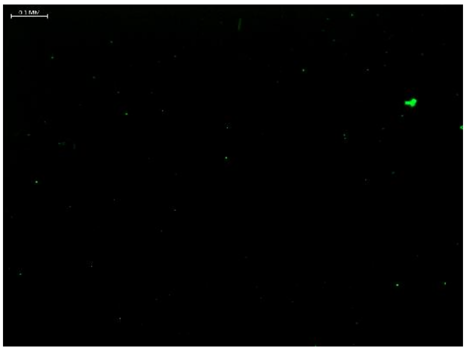

Bil-PI

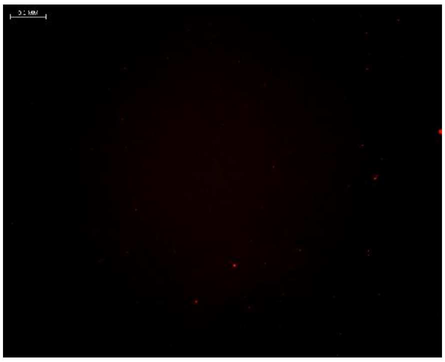

Bil-Merge

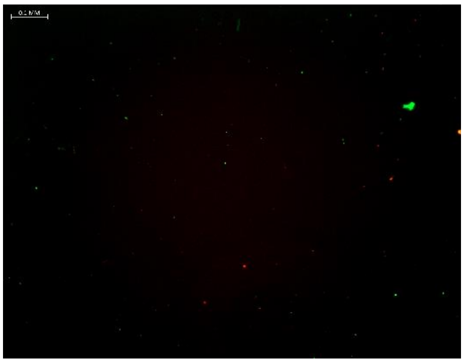

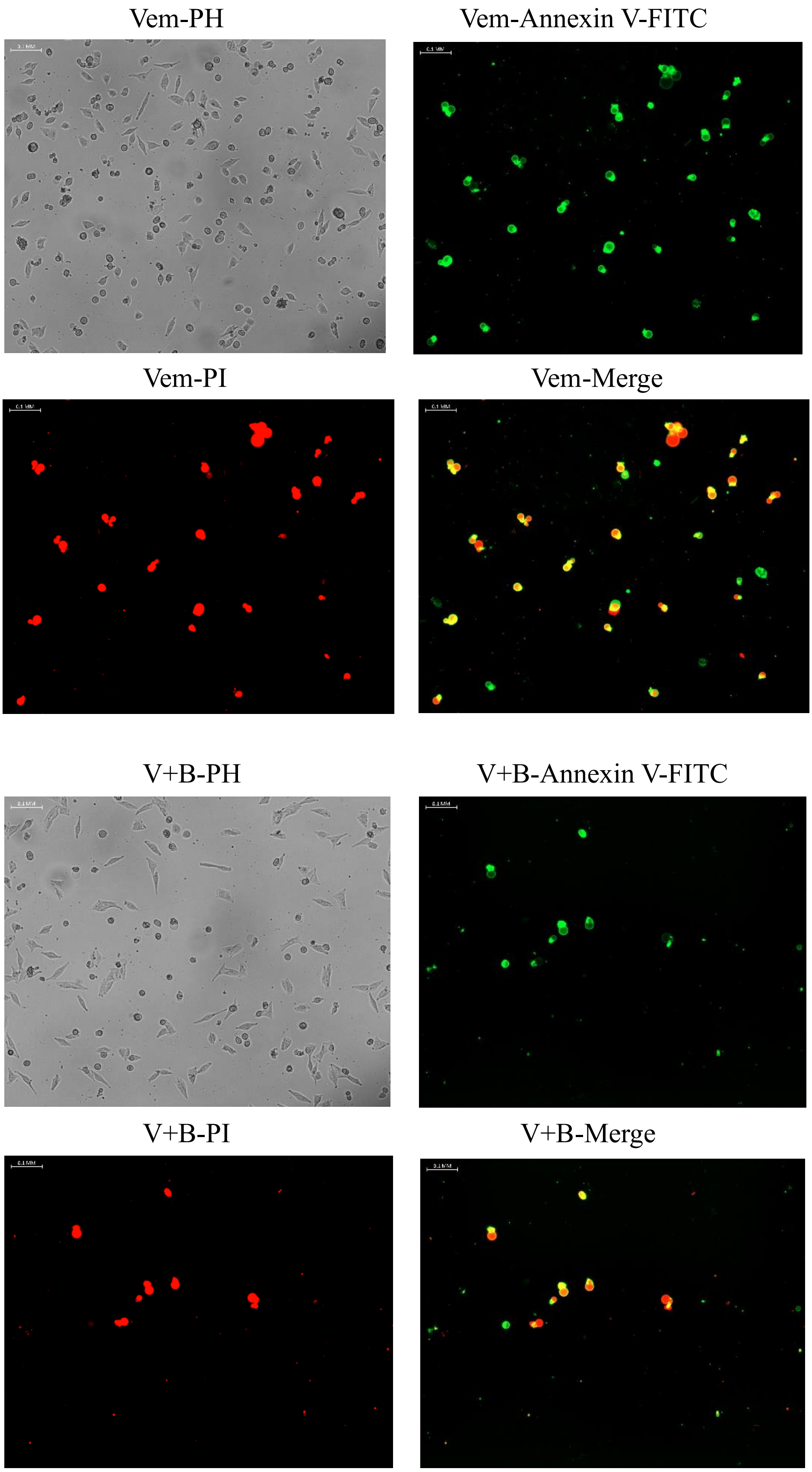
Vem-PH
Vem-Annexin V-FITC
Vem-PI
Vem-Merge
V+B-PH
V+B-Annexin V-FITC
V+B-PI
V+B-Merge
