# Supplementary material for: Bilirubin Restrains the Anticancer Effect of Vemurafenib on BRAF-Mutant Melanoma Cells Through ERK-MNK1 Signaling
Source: Front Oncol. 2021 Jun 18;11:698888. doi: 10.3389/fonc.2021.698888 (PMC8250144; doi:10.3389/fonc.2021.698888)
Supplement: Supplementary file 2 [file Table_1.docx]

**Supplementary Table 1.** Correlation between age/gender and clinical outcome of patients with BRAF mutant melanoma.

**Table 1**

**Clinical Outcome *P*-value**

**Clinicopathological Features PD SD PR CR**

Age

＜60 3 (4.1) 10 (10.7) 10 (8.2) 0 0.130

≥60 2 (0.9) 3 (2.3) 0 (1.8) 0

Gender

Male 3 (2.5) 7 (6.5) 4 (5.0) 0 0.713

Female 2 (2.5) 6 (6.5) 6 (5.0) 0
